# Mixed T-Cell Chimerism Following Hematopoietic Cell Transplantation for Non-Malignant Disorders Is Common, Facilitates Anti-Viral Immunity, and Is Not Associated with Graft Failure in Pediatric Patients

**DOI:** 10.3390/cells13242119

**Published:** 2024-12-20

**Authors:** Rubiya Nadaf, Helena Lee, Denise Bonney, Ramya Hanasoge-Nataraj, Srividhya Senthil, Claire Horgan, Malcolm Guiver, Kay Poulton, Robert Wynn

**Affiliations:** 1 Departments of Blood and Marrow Transplant, Royal Manchester Children’s Hospital, Manchester M13 9WL, UK; denise.bonney@mft.nhs.uk (D.B.); ramya.hanasoge-nataraj@mft.nhs.uk (R.H.-N.); srividhya.senthil@mft.nhs.uk (S.S.); claire.horgan@mft.nhs.uk (C.H.); 2 Departments of Transplantation Laboratories, Manchester Royal Infirmary, Manchester M13 9WL, UK; helena.lee@mft.nhs.uk (H.L.); kay.poulton@mft.nhs.uk (K.P.); 3 Departments of Virology Laboratories, Manchester Royal Infirmary, Manchester M13 9WL, UK

**Keywords:** mixed T-cell chimerism, hematopoietic cell transplantation (HCT), non-malignant disorders, anti-viral immunity, chimerism

## Abstract

Myeloid chimerism better reflects donor stem cell engraftment than whole-blood chimerism in assessing graft function following allogeneic hematopoietic stem cell transplant (HCT). We describe our experience with 130 patients aged younger than 18 years, treated with allogeneic HCT using bone marrow or PBSC from HLA-matched donors for non-malignant diseases, whose pre-transplant conditioning therapy included alemtuzumab and who were monitored with lineage-specific chimerism after transplant. At 6 years post-transplant, overall survival (OS) was 91.1% and event-free survival (EFS) was 81.5%, with no grade III-IV acute GvHD or chronic GVHD observed. Recipient T-cells did not contribute to graft loss. Mixed T-cell chimerism (MC) did not affect EFS, and there was no connection between T-cell chimerism and myeloid chimerism in patients with MC or graft loss. MC significantly correlated with virus infection; more children with MC were CMV seropositive than those with complete chimerism (CC). Additionally, MC was more common in patients with CMV viramia post-transplant. CD8 T-cell reconstitution was affected by viral reactivation, including CMV, with CD8 T-cell counts higher in the MC group than in the CC group. Mixed T-cell chimerism is due to autologous, virus-specific, predominantly CD8, T-cell expansion, and is protective and not deleterious to the recipient.

## 1. Introduction

Hematopoietic stem cell transplantation (HSCT) is a treatment with curative potential for many non-malignant diseases (NMD) [1]. Post-transplant surveillance and interventions, which are critical to transplant success, include chimerism analysis. Chimerism analysis is performed to evaluate the donor engraftment status and resolution of the NMD [2]. Myeloid chimerism is a more accurate indication of stem cell engraftment following transplant (HCT) than T-cell chimerism since myeloid cells have a shorter life span and therefore better reflect current hematopoiesis [3]. The significance of chimerism after HCT for non-malignant disease (NMD) in children remains uncertain. In a spectrum of non-malignant hematologic disorders, it is not a requisite to completely substitute the patient’s hematopoietic and immune systems with donor cells. This is evidenced by clinical studies indicating successful patient outcomes without achieving full donor chimerism [4].

Engraftment is achieved by both the pre-transplant depletion of recipient stem cells during conditioning chemotherapy and the post-transplant rejection of residual recipient HSC by engrafted donor immune cells. As such, incidence of mixed chimerism (MC) is influenced by the intensity of conditioning and is increased in NMD when there are reduced intensity conditioning (RIC) regimens during transplant [5,6]. Donor chimerism (DC) is favored by the immune action of the engrafted donor cells [7] and is higher in patients with graft versus disease (GVHD) [8], including after donor lymphocyte infusion (DLI) [9], and is reduced where donor T-cells are depleted from the graft [10]. Immune reconstitution and chimerism patterns are significantly influenced by the involvement of cytomegalovirus (CMV)-specific T-cells [11]. Autologous CMV-specific T-cells might expand during post-transplant CMV reactivation, generating mixed T-cell chimerism but protecting the patient from the virus [12]. In malignant disease, strategies to promote DC are beneficial since the occurrence of mixed T-cell chimerism has been shown, most recently by Craddock et al., to be associated with an increased risk of disease relapse after HCT [13]. Its prevention by myeloablative (MAC) conditioning [14], or its reversal by either donor lymphocyte infusion (DLI) or rapid tapering of pharmacological immune suppression, reduces the risk of treatment failure [15].

Although understanding the implications of MC after hematopoietic cell transplantation (HCT) in pediatric NMD is crucial, the interpretation of its significance remains uncertain. Certain studies indicate that MC may lead to a heightened risk of graft failure and disease recurrence [16]. Conversely, there are reports that suggest the possible existence of stable MC, without any adverse effects on long-term prognosis. [4]. The aim of HCT in NMD is disease-free, GVHD-free, drug-free, long-term survival. Interventions to reverse MC, such as DLI and manipulation of immune suppression, threaten this aim in an individual patient. We retrospectively reviewed lineage-specific chimerism to understand its relevance to graft and patient outcomes in children with NMD undergoing T-cell depleted HCT from matched sibling or unrelated donors.

## 2. Materials and Methods

With specific consent to use their transplant data for research and registry purposes, the medical records of 141 patients aged younger than 18 years who underwent allogeneic, HLA-matched HCT using a stem cell source other than cord blood for the non-malignant disorder at Royal Manchester Children’s Hospital between September 2014 to May 2020 were reviewed. The analysis included all patients diagnosed with benign hematologic and immunological disorders. Patients who died within three months following HSCT and those who experienced primary graft failure were excluded from the study.

### 2.1. Graft Source

The types of grafts utilized include human leukocyte antigen (HLA)-matched or 1 antigen mismatched grafts from related or unrelated donors, either with bone marrow (BM) or peripheral blood (PBSC) as stem cell source (PBSCs).

### 2.2. Conditioning Regimens

Myeloablative (MAC) and reduced intensity (RIC) protocols were utilized in patients. MAC was given to patients with metabolic disease, hemoglobinopathies, and certain immunodeficiency disorders. Fludarabine was administered in combination with pharmacokinetic (PK)-guided intravenous busulfan (Flu-Bu), aiming to achieve a busulfan exposure of 80–90 mg/h/L plasma concentration–time curve (AUC), or it was given alongside Thiotepa (10 mg/kg in 2 divided doses) and Treosulfan (42 gms/m2, provided as 3 daily doses) (FTT). All patients with inherited bone marrow failure syndrome and certain immunodeficiencies received RIC with fludarabine, either with busulfan targeting an AUC of less than 65 mg/h/L, or with cyclophosphamide.

All patients received GVHD prophylaxis with in vivo T-cell depletion with alemtuzumab [17]. Family donor transplants received cumulative dose of 0.3 mg/kg, administered as 3 equal successive daily doses, and unrelated donor transplants received cumulative dose of 1 mg/kg, administered as 5 equal successive daily doses. The last day of alemtuzumab administration was day -5 in all cases. Intravenous ciclosporin (CsA) was given as post-transplant GVHD prophylaxis at a loading dose of 3 mg/kg starting at day-3 to -1, followed by a maintenance dose of 2.5 mg/kg. The CSA doses were adjusted to achieve a trough level between 100 and 200 ng/mL. Additional prophylaxis with mycophenolate mofetil (MMF) was used in patients receiving grafts from mismatched donors, or those in whom the stem cell source was mobilized peripheral blood. MMF was given at a dose of 15 mg/kg three times daily starting at day 0. In the absence of GVHD, ciclosporin was tapered from 6 months after HSCT over a period of 2–4 weeks. When used, MMF was weaned from day 28 after HSCT and stopped over 2 weeks in the absence of GVHD. Immune suppression was weaned regardless of donor T-cell chimerism.

### 2.3. Chimerism Assessment

Whole-blood (WB) chimerism was measured in all patients at engraftment, monthly until 6 months, and at 9 and 12 months after transplant. Where WB chimerism was mixed, T-cell and myeloid lineage-specific chimerism was measured. Where WB chimerism was fully donor, T-cell and myeloid chimerism were assessed as fully donor. Chimerism was evaluated using short-tandem repeat polymerase chain reaction (STR-PCR), which was performed using Gene Print^®^. A DNA profile was generated for the recipient and donor, which was used for subsequent post-transplant chimerism analysis. A minimum of three informative markers was required for successful analysis. Where lineage-specific chimerism was performed, CD3 T-cells and CD15 myeloid cells were isolated from the peripheral blood by positive selection magnetic-activated cell sorting (MACS) kits using the RoboSep™-S instrument.

### 2.4. Definitions of Hematological Engraftment, Chimerism States, and Graft Failure

Neutrophil Engraftment was defined as a sustained peripheral blood neutrophil count of >0.5 × 10^9^/L or more for 3 consecutive days [18]. Platelet engraftment was defined as independence from platelet transfusion for at least 7 days, and with a platelet count of more than >20 × 10^9^/L [18].

Complete chimerism was defined as greater than 95% of the donor whole blood (WB) and/or CD15 Myeloid cells on STR analysis post engraftment [19,20]. Mixed chimerism (MC) was defined as the presence of less than 95% donor WB or myeloid cells at any time after HSCT [19,20].

Graft failure (GF) comprises primary graft failure and autologous reconstitution. Primary graft failure is defined as no evidence of engraftment or hematologic recovery of donor cells by Day + 30 [21]. Primary graft failure is considered immune-mediated [22]. Autologous reconstitution is the later replacement of donor cell hematological engraftment by recipient-cells and is defined as myeloid engraftment of less than 20% by STR analysis.

### 2.5. Disease Response to Transplant

Since there are no clear criteria on the definition of remission in patients with non-malignant diseases, we assessed the disease response to the transplant using disease-specific measures. These included transfusion independence and the restoration of normal full blood counts in patients with bone marrow failure syndromes; immune reconstitution and the resolution of infections in patients with immune deficiencies; enzyme correction in patients with metabolic disorders; and the absence of symptoms and the discontinuation of pre-transplant specific therapies in patients with autoimmune disorders.

### 2.6. CMV Serology and Reactivation

Pre-transplant CMV serology was recorded. Patients were screened weekly for CMV viremia using PCR, and a single result of more than 1000 copies/mL, or 2 results with more than 500 copies/mL, was regarded as CMV viremia.

### 2.7. Graft versus Host Disease (GVHD)

Acute graft-versus-host disease (aGVHD) was classified and graded with the method of Glucksberg grading [23].

### 2.8. Statistical Analysis

All statistical analyses were conducted using GraphPad Prism 9.0. Statistical models were utilized to investigate the relationships between CD3 and CD15 donor chimerism, patient- and transplant-associated factors, and graft outcomes. The Kaplan–Meier method was employed to estimate the probabilities of overall survival (OS) and event-free survival (EFS). Survival rates were compared using the log-rank test (Mantel–Cox). In the scope of this study, an “event” was specifically defined as the occurrence of either grade 3–4 graft-versus-host disease (GVHD), death, or a relapse of the disease. Logistic regression was applied to correlate transplant variables with chimerism in both univariate and multivariate analyses. The correlation between CD3 and CD15 values at various time points was determined using Pearson’s correlation coefficient (r). The graphical representation of chimerism measurement was compiled using the mean at each time point, separately for whole-blood and lineage-specific measurements. When comparing more than two groups of means, the ordinary one-way ANOVA test was applied, and the unpaired t-test was used in other cases. Group comparison was performed using non-parametric tests, namely, Pearson’s Chi-square and Fisher’s exact tests. A *p*-value of less than 0.05 was deemed significant.

## 3. Results

### 3.1. Transplant Characteristics

The characteristics of patients and transplants are presented in Table 1**.** This study comprised 141 patients, among whom 118 patients exhibited sustained donor-derived engraftment, 12 patients experienced late graft failure, and 11 patients died. Survival curves were analysed using a total of 141 patients, and the rest of the analysis was limited to the 130 patients who survived long-term. This group consisted of 55 females and 75 males with a median age of 5.2 years, with ages ranging from 0.1 to 18 years.

NMD diagnosis included metabolic disorders (*n* = 34); immunological disorders, including immunodeficiency diseases (*n* = 36); hematological conditions, including hemoglobinopathies (*n* = 19); and aplastic anemia (*n* = 30), itself including inherited bone marrow failure syndromes (*n* = 41). All donor–recipient pairs underwent high-resolution HLA-typing at class I (HLA-A, -B, and -C,) and class II (HLA-DR and –DQ). The donors for these 130 transplants included 10/10 matched unrelated donors (MUDs, *n* = 53), mismatched unrelated donors (mMUDs, *n* = 15), matched sibling donors (MSDs, *n* = 45), matched family donors (MFDs, *n* = 8), and mismatched family donors (mMFDs, *n* = 9). The 24 mismatched donors were mismatched at a single antigenic locus. The source of the graft was either bone marrow (BM, *n* = 111) or peripheral blood stem cells (PBSC, *n* = 19). MAC Flu-Bu was given to 27 patients, and MAC FTT was given to 64 patients. RIC was used in 39 patients. Univariate and multivariate analyses of transplant characteristics, which include age at transplant, primary diagnosis, donor type, HLA disparity, stem cell source, and intensity of conditioning, were not predictive of T-cell chimerism or graft outcome.

### 3.2. Alemtuzumab in Conditioning Is Associated with High Rates of Engraftment, and Low Rates of Acute and Chronic GVHD

**Figure 1:**
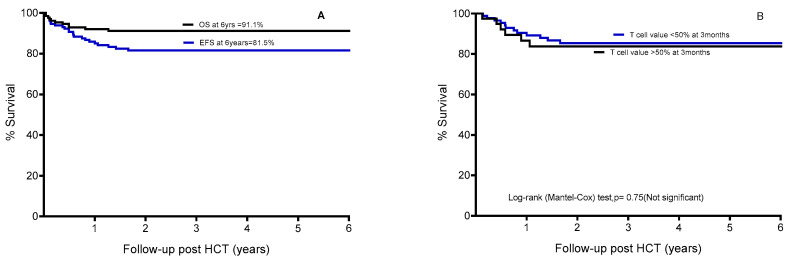
(**A**) Kaplan–Meier curves showing overall survival (OS) and event-free survival (EFS). An event was defined as graft failure, death, or chronic GVHD requiring immune suppression. (**B**) Kaplan–Meier curves showing event-free survival in two groups: T-cell chimerism less than 50%, *n* = 35; and T-cell chimerism more than 50% at 3 months, post-HCT, *n* = 95. Study cohort *n* = 130, Log-rank (Mantel–Cox) test, *p* = 0.75.

The median follow-up period post-transplant was 2.8 years. The overall survival (OS) and engrafted, GVHD-free survival (EFS) rates six years post-transplant were 91.1% and 81.5%, respectively (Figure 1A). There were no instances of grade III–IV acute GvHD, nor were any cases of chronic GVHD noted.

There were no cases of primary graft failure. Seventy-three patients (56.1%) had CC, while forty-five (34.6%) exhibited MC, and twelve patients experienced late GF with late autologous reconstitution. Of these 12 patients, 8 were successfully re-transplanted at a median time of 12 months after their first transplant. All patients who underwent re-transplantation maintained adequate donor cell engraftment, and in most cases, the same donor was used.

### 3.3. T-cell Chimerism Does Not Predict for Graft Loss

The presence of early mixed T-cell chimerism did not predict graft loss. The event-free survival (EFS) and engrafted survival of those with early donor T-cell chimerism (CD3+)—either below or above 50% at 3 months—are the same (Figure 1B), indicating that mixed T-cell chimerism impacts neither engrafted survival nor overall survival.

**Figure 2:**
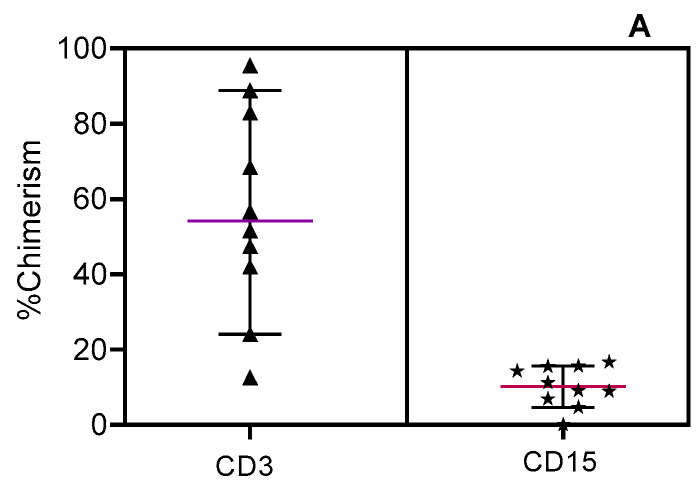

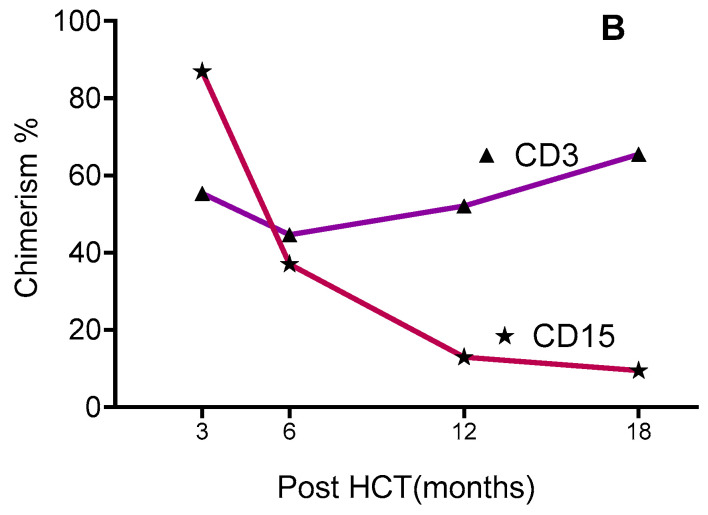
(**A**) Comparison of last recorded lineage-specific chimerism data of the graft failure group; *n* = 12. *p*-value determined via unpaired two-tail *t*-test, *p* = 0.0001. (**B**) CD3+/CD15+ trends in chimerism values in the graft loss group over time.

In those that experience GF, the mean T-cell (CD3+) donor chimerism and mean myeloid (CD15+) donor chimerism at graft loss are 57.02% and 10.28%, respectively, and there is no correlation between the T-cell and myeloid chimerism (Figure 2A). Indeed, throughout the decline of myeloid chimerism that characterizes GF, there was an observable increase in T-cell chimerism (Figure 2B).

### 3.4. In Patients with Mixed T-Cell Chimerism, Donor T-Cell Chimerism Rises with Time from Transplant and Is Correlated Neither with Myeloid Chimerism Nor with GF

**Figure 3:**
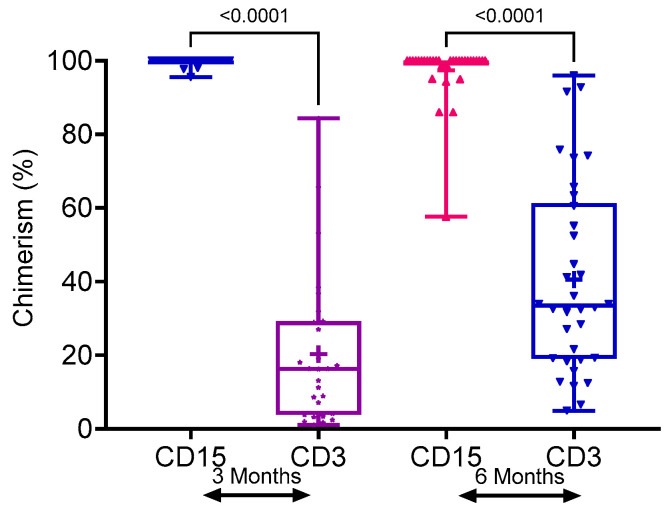
Box plots of comparison of lineage-specific chimerism in stable mixed chimerism groups.

At 3 and 6 months post-HCT, the mean T-cell (CD3) and myeloid (CD15) donor chimerism values varied significantly. In the mixed WB chimerism group, these were 20.28% and 99.60% at 3 months, and 40.55% and 97.34% at 6 months, respectively (*p* < 0.0001) (Figure 3). In most patients who exhibited MC, myeloid (CD15) donor chimerism remained fully donor. This suggests that mixed whole blood (WB) chimerism reflects mixed T-cell chimerism. On the other hand, we note that T-cell chimerism mean rises with time in children who are donor cell engrafted, as the graft makes T-cells.

Moreover, Pearson’s correlation test showed no correlation between donor CD3+ and CD15+ chimerism at the 3, 6, and 12-month post-transplantation. In those with mixed T-cell chimerism, there is no relationship between T-cell chimerism and eventual graft loss. An unpaired two-tailed t-test in the mean T-cell chimerism at 3 months is similar in the MC and GF groups (unpaired 2-tailed *t*-test, *p* = 0.11).

### 3.5. CMV Positive Serology and CMV Viremia and Accelerated CD8 Immune Reconstitution Are Commoner in the MC Cohort Compared with the CC Cohort

CMV viremia was observed in 31.8% and was significantly more common in CMV seropositive patients (R+) than in seronegative patients (*p* ≤ 0.0001).

**Figure 4:**
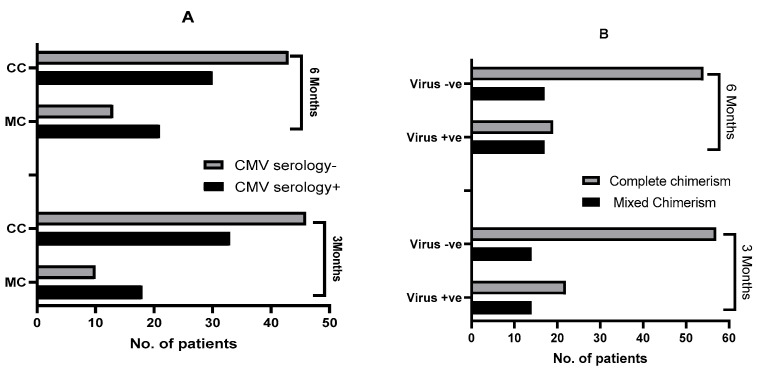
(**A**) Impact of CMV serology in relation to chimerism status at 3 and 6 months post-HCT. Fisher’s exact test was used to compute the group contingencies; n = 107. At 3 months, in the MC group, 64.2% (18/28) were CMV seropositive (R+) and 35.7% (10/28) were CMV seronegative (R−). At 3 months, in the CC group, 41.7% (33/79) were R+ and 58.2% (46/79) were R−; *p* = 0.049 via Chi-square test. At 6 months, in the MC group, 61.7% (21/34) were CMV seropositive (R+) and 38.2% (13/34) were CMV seronegative (R−). At 6 months, in the CC group, 41.1% (30/73) were R+ and 58.9% (43/73) were R−; *p* = 0.04 via Chi-square test. There is a statistically significant relationship. (**B**) Impact of CMV viremia in relation to chimerism status at 3 and 6 months post-HCT. At 3 months, in the MC group, 50% (14/28) had CMV viremia and 50% (14/28) were negative. At 3 months, in the CC group, 27.8% (22/79) had CMV viremia and 72.1% (57/79) were negative. At 6 months, in the MC group, 50% (17/34) had CMV viremia and 50% (17/34) were negative. At 6 months, in the CC group, 26% (19/73) had CMV viremia and 73.9% (54/73) were negative; *p* = 0.03 and 0.01 by Chi-square test.

CMV seropositivity was more frequent among the mixed T-cell chimerism group. CMV positive serology was found in 64% (18/28) and 61.7% (21/34), respectively, in the mixed T-cell chimerism group at 3 and 6 months, compared with 41% of the CC at both of these time points (33/79 and 30/73) (*p* < 0.05) (Figure 4A).

Similarly, CMV viremia was significantly more prevalent in the MC group. At 3 and 6 months, the reported incidence of viremia in the MC group was 50%, compared to 28% and 26% in the complete chimerism group (*p* = 0.03 and 0.01, respectively) (Figure 4B).

**Figure 5:**
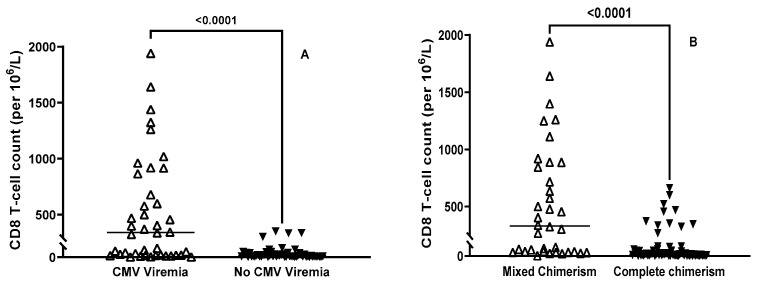
(**A**) Distribution of CD8 T-cell count in CMV-positive and -negative patients at 3 months post-HCT. Mean CD8 T-cell was 548 in the CMV viremia group and 77.7 in patients with no CMV infection; *p* = 0.0001. (**B**) Distribution of CD8 T-cell count in the mixed chimerism and complete chimerism groups at 3 months post-HCT.

There was a significant increase in the CD8 T-cell count within the CMV viremic cohort compared to those without CMV viremia (mean of 548 vs. 77.7, *p* = 0.0001, at 3 months; mean of 780 vs. 244, *p* ≤ 0.0001, at 6 months) (Figure 5A). We compared CD8 T-cell reconstitution within the complete chimerism (CC) and the mixed chimerism (MC) groups. At 3 months post-HCT, the MC cohort had higher CD8 T-cell counts than the CC group (mean of 402 vs. 153, *p* = 0.005) (Figure 5B).

## 4. Discussion

These data describe excellent GVHD-free, disease-free survival with low transplant-related mortality in children with NMD and with MAC and RIC protocols, including in vivo T-cell depletion with alemtuzumab. Mixed chimerism is common in our patient cohort, principally reflecting mixed T-cell chimerism. We demonstrate that there is no relationship between this mixed T-cell chimerism and subsequent graft loss. The presence of mixed T-cell chimerism did not influence EFS, there was no relationship between T-cell chimerism and myeloid chimerism, and in those patients with graft loss, there was an increase in recipient chimerism during decline in myeloid chimerism.

We note that those children with mixed T-cell chimerism are more commonly those that are CMV seropositive, or those that have experienced a CMV reaction. CD8 immune reconstitution is accelerated in the MC group compared to the CC group, as well as in those with CMV viremia. These data indicate that the mixed T-cell chimerism is secondary to the expansion of residual, autologous, virus-specific, likely CD8 T-cells (CMV, but likely other viruses too), and is protective to the recipient. Sellars et al. previously described that the mixed T-cell chimerism after reduced-intensity, alemtuzumab-based conditioning in CMV-positive recipients of a CMV seronegative graft was similarly due to the expansion of autologous CMV specific recipient T-cells [11,12]. They were additionally able to demonstrate recipient CMV specificity using multimer analysis [11,12]. This expansion of residual recipient T-cells occurs in our cohort even following myeloablative conditioning. Donahue [24] describes significant failure of recipient T-cell depletion from tissues in a primate model of a myeloablative transplant, and we propose that we are describing a similar phenomenon in our patient cohort.

Graft failure is a failure of myelosuppression rather than a failure of immune suppression [25]. Strategies to promote DC, including escalation of conditioning intensity, will increase transplant-related mortality (TRM) and transplant late effects [26], end with DLI promotion of the immune clearance of residual recipient-cells by the engrafted donor-derived immune system will risk GVHD [27], which greatly impacts one’s long-term quality of life [28] and is not directly beneficial to the underlying disease, itself risking TRM.

**Figure 6:**
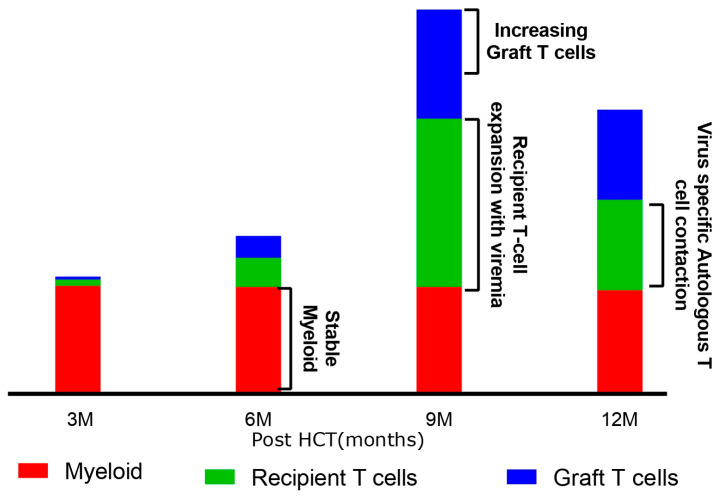
Summative figure showing autologous T-cell expansion with viral infection.

Figure 6 illustrates this model of mixed chimerism following transplant in NMD in children.

- Myeloid engraftment reflects stem cell engraftment and is maintained except in those that suffer graft failure with autologous reconstitution. Myeloid chimerism is much more important to the clinician monitoring engraftment after NMD transplant.
- As donor stem engraftment is sustained, the cumulative graft output of donor-derived T-cells is increased.
- In the early post-transplant period then residual recipient, virus-specific autologous T-cells might expand and dilute these graft-derived T-cells. This is the basis of mixed T-cell chimerism after transplant.

Importantly, the significance of mixed T-cell chimerism is different than after malignant disease transplant. In the latter, the failure of engrafted donor T-cells to eradicate recipient T-cells—already likely deleted by rounds of pre-transplant chemotherapy—indicates a likelihood that those donor T-cells will also fail to eradicate residual host leukemia. This immune action of the donor T-cells to control disease and prevent disease relapse in leukemia can be fortified with additional infusions of donor T-cells [29].

We neither intensified immune suppression nor used DLI in our patients with MC after HSCT for NMD. Where the MC is due to mixed T-cell chimerism, we have shown that this is likely associated with viral infection control and is not associated with graft loss, so intervention is unnecessary and potentially harmful if it removes an effective viral immune response. Whilst DLI might eradicate recipient hematopoiesis during mixed myeloid chimerism after HSCT, DLI are significantly associated with chronic GVHD, and this is of no benefit to the child with NMD. Where GF occurs, we have preferred to use second HSCT to restore donor hematopoiesis and correct the underlying disease since GVHD rates will be low.

Our study, however, is not without limitations. It was specifically focused on HLA-matched patients and donors receiving BM or PBSC grafts and those that received in vivo T-cell depletion with alemtuzumab. Its findings might not extend to those transplanted under other conditions, including those receiving haplo-identical or CB grafts.

## Figures and Tables

**Table 1:** Univariate analysis of pre-transplant factors as a predictor for alive stable engraftment/graft loss.

	Engrafted	Graft Loss	OR	95%CI	*p*-Value
Number of patients	118	12			
Pre-transplant factors					
Sex, n (%)					
Male	61	7	0.9	0.31–2.9	0.9
Female	57	5			
Age at transplant, years					
Median	5.2	3.5			
Range	0.3–18.6	0.1–17			
Diagnosis					
Aplastic	28	2			0.5
Metabolic	30	4			
Hematological	28	2			
Immunological	32	4			
Aplastic	28	2	1.7	0.43–8.4	0.7
Non-aplastic	90	10			
Donor					
Family	55	5	1.32	0.4–3.9	0.76
Unrelated	63	7			
HLA disparity					
Matched (10/10)	95	11	1.29	0.10–9.0	0.58
Mismatched (9/10)	23	1			
Stem cell source					
PBSC	17	2	0.94	0.2–4.6	0.99
BM	101	10			
Conditioning					
MAC-Bu-Flu	25	2			0.17
MAC-FTT	55	9			
RIC	38	1			

## Data Availability

Data are available on request.
